# Mutation of the Fiber Shaft Heparan Sulphate Binding Site of a 5/3 Chimeric Adenovirus Reduces Liver Tropism

**DOI:** 10.1371/journal.pone.0060032

**Published:** 2013-04-09

**Authors:** Anniina Koski, Eerika Karli, Anja Kipar, Sophie Escutenaire, Anna Kanerva, Akseli Hemminki

**Affiliations:** 1 Cancer Gene Therapy Group, Molecular Cancer Biology Program and Transplantation Laboratory and Haartman Institute, University of Helsinki, Helsinki, Finland; 2 Finnish Centre for Laboratory Animal Pathology, Faculty of Veterinary Medicine, University of Helsinki, Helsinki, Finland; 3 Veterinary Pathology, School of Veterinary Science and Department of Infection Biology, Institute of Global Health, University of Liverpool, Liverpool, United Kingdom; 4 Department of Obstetrics and Gynecology, Helsinki University Central Hospital, Helsinki, Finland; Mayo Clinic, United States of America

## Abstract

Natural tropism to the liver is a major obstacle in systemic delivery of adenoviruses in cancer gene therapy. Adenovirus binding to soluble coagulation factors and to cellular heparan sulphate proteoglycans *via* the fiber shaft KKTK domain are suggested to cause liver tropism. Serotype 5 adenovirus constructs with mutated KKTK regions exhibit liver detargeting, but they also transduce tumors less efficiently, possibly due to altered fiber conformation. We constructed Ad5/3lucS*, a 5/3 chimeric adenovirus with a mutated KKTK region. The fiber knob swap was hypothesized to facilitate tumor transduction. This construct was studied with or without additional coagulation factor ablation. Ad5/3lucS* exhibited significantly reduced transduction of human hepatic cells *in vitro* and mouse livers *in vivo*. Combination of coagulation factor ablation by warfarinization to Ad5/3lucS* seemed to further enhance liver detargeting. Cancer cell transduction by Ad5/3lucS* was retained *in vitro*. *In vivo,* viral particle accumulation in M4A4-LM3 xenograft tumors was comparable to controls, but Ad5/3lucS* transgene expression was nearly abolished. Coagulation factor ablation did not affect tumor transduction. These studies set the stage for further investigations into the effects of the KKTK mutation and coagulation factor ablation in the context of 5/3 serotype chimerism. Of note, the putative disconnect between tumor transduction and transgene expression could prove useful in further understanding of adenovirus biology.

## Introduction

Adenoviral vectors are a promising novel approach for cancer gene therapy. In clinical trials, intratumoral injections of adenoviruses have shown promising efficacy [1], [2], [3]. However, in the case of widely metastatic cancers, efficient systemic delivery would be attractive. Despite some evidence of antitumor activity, the efficacy of intravenously administered adenovirus has so far not been optimal [4], [5]. One of the major hurdles in systemic delivery of adenoviruses is the natural liver tropism of the most commonly used serotype 5 adenovirus (Ad5) vector and its derivatives. After systemic administration, the majority of virus particles rapidly accumulate in the liver, which is also the major site of gene expression [6], [7], [8], [9]. Liver interactions also contribute to adenoviral toxicity. Elevations of liver transaminases as a sign of acute liver toxicity are frequently observed following systemic delivery of adenoviruses [10], [11]. Systemic administration of adenovirus vectors can also cause toxicity through induction of inflammatory cytokines, triggered by activation of antigen-presenting cells, such as Kupffer cells and tissue macrophages, in liver and spleen [12], [13], [14], [15], [16], [17].

Adenoviral binding *via* heparan sulphate proteoglycans (HSPGs) has been hypothesized to play an important role in liver transduction both in the presence and absence of the primary receptor CAR [18], [19]. The region responsible for HSPG binding is thought to be located at the KKTK motif in the third repeat of the adenoviral fiber shaft [18], although this has not been clearly proven. Nevertheless, modification of this Ad5 shaft region has been described to profoundly influence liver transduction in mice, rats and non-human primates [20], [21], [22], [23], [24]. These effects have been even more pronounced when viruses do not present a tropism for CAR. Also, cytokine responses and liver enzyme elevations have been less pronounced with KKTK mutated vectors both in mice and non-human primates [20], [25].

Unfortunately, transduction of target tissues, including tumors, has been in efficient with the Ad5 based KKTK mutated vectors, which has limited the utility of the approach thus far [20], [21], [25], [26]. Moreover, even if these vectors in theory offer a perfect backbone to target adenovirus tropism to the receptor of choice by inserting new ligands, the vectors have in fact not been efficiently retargetable. Insertion of targeting ligands to the HI loop of KKTK mutated viruses has been able to restore transduction *in vitro* to some extent [23], [25], [26], but this could not be reproduced *in vivo* [26]. One hypothesis as to why the transduction with KKTK mutants is reduced in such a wide range of tissues is that the mutation not only disturbs HSPG binding, but also impairs the flexibility of the shaft, as a region providing shaft flexibility also resides in the same locus [21], [27]. Thus, the long Ad5 shaft becomes rigid, which may prevent interactions with other cellular virus binding motifs, including the fiber knob, by sterically inhibiting the virus from interacting with the cell surface.

For Ad5, fiber length and flexibility have been shown to be essential for cell interactions and entry, possibly by allowing the virus to overcome charge-dependent repulsion between the virus capsid and the acidic cell surface [27], [28]. In contrast, Ad3 is a species B adenovirus with a notably shorter fiber than Ad5, consisting of only 6 shaft repeats compared to the 22 repeats of Ad5 [29]. The short shafted Ad3also lacks the KKTK region of the third repeat [18] and may therefore be less dependent on fiber flexibility. Serotype 5 adenoviruses with chimeric fibers carrying the serotype 3 knob employ alternate cell binding properties [30], [31]. Even before the primary receptor for Ad3 was discovered, these Ad5/3 chimeras were shown to exhibit enhanced gene delivery and antitumor efficacy in preclinical assays with cell lines, fresh clinical specimens and animal models [8], [32], [33], [34], [35], [36], [37], [38]. Thereafter, CD80 and CD86 and desmoglein-2 (DSG-2) have been suggested as possible receptors for Ad3 [39], [40]. CD46 and HSPGs have also been identified as having minor contributions in Ad3 infection [41], [42]. Currently, the majority of publications seem to agree that DSG-2 is the primary receptor for Ad3, at least *in vitro* [42], [43]. Fully Ad3 viruses do not infect rodent cells [40], and therefore transgenic mice expressing human DSG-2 have also been developed for further studies of Ad3 [44]. However, although apparently able to utilize DSG-2 as receptor, the Ad5/3 chimeras are not completely dependent on DSG-2 even *in vitro*, as demonstrated by less efficient blocking of infection by recombinant DSG-2 in competitive assays [40]. Further, *in vivo* Ad5/3 chimeras seem to not depend on expression of human DSG-2 as they have been shown to efficiently transduce mice tissues to similar levels as Ad5 [8], [34] and they can also deliver transgenes for achieving antitumor efficacy in syngeneic murine tumor models [45].

In addition to direct binding between virus and receptors, soluble factors present in the circulation have been shown to be important mediators of adenoviral tissue transduction. Multiple vitamin K dependent coagulation factors, factor IX (FIX) and factor X (FX) in particular, have been shown to bind Ad5 and mediate hepatocyte transduction [17], [46], [47]. Interestingly, interaction with these coagulation factors has been demonstrated to be particularly important for liver transduction [46], [48], [49], [50].

The aim of this study was to construct and test a chimeric adenovirus with a type 5 backbone, a type 3 knob and a KKTK mutation in the Ad5 fiber shaft. The mutation of the KKTK region is expected to detarget the virus from the liver and Ad3 knob is expected to allow for retained tumor transduction. As native Ad3 does not require a long flexible shaft for cell interaction and entry, the Ad5/3 chimera might also tolerate the loss of flexibility caused by mutation of the KKTK region. In addition, we aimed to study the effect of coagulation factors on gene expression by this mutated chimeric virus, and to compare liver detargeting by KKTK mutation to liver detargeting via coagulation factor ablation. As Ad5/3 chimeric viruses have been demonstrated to deliver transgenes efficiently also in mouse tissues, which lack human DSG-2, we studied these modifications in a human xenograft mouse model, so that the results could be easily compared with previous findings concerning the KKTK mutated viruses and warfarinization.

## Materials and Methods

### Ethics Statement

All animal protocols were reviewed and approved by the Experimental Animal Committee of the University of Helsinki and the Provincial Government of Southern Finland.

### Cell Lines

293 transformed human embryonic kidney cells and PC-3 prostatic carcinoma cells were obtained from the American Type Culture Collection (ATCC; Manassas, VA, USA), M4A4-LM3 [51] human breast cancer cells were donated by Prof. Pirjo Laakkonen (University of Helsinki, Helsinki, Finland), Hey human ovarian adenocarcinoma cell line, originally from ATCC, and SKOV3.ip1 human ovarian adenocarcinoma cell line [52] were donated by Drs Judy Wolf and Janet Price (both M. D. Anderson Cancer Center, Houston, Texas, USA), and the HepG2 human hepatocellular carcinoma cell line, originally from ATCC, was donated by Sanna Toivonen (University of Helsinki, Helsinki, Finland). All cells were maintained and propagated as recommended. Growth media were supplemented with 10% fetal calf serum (FCS), 1% L-glutamine and 1% penicillin-streptomycin antibiotics. Virus infections were carried out in media supplemented with 2% FCS.

### Viruses

Ad5/3luc1 and Ad5luc1 have been previously described [31], [53]. Ad5/3luc1 has a chimeric fiber protein with knob from serotype 3 replacing the native Ad5 knob protein. All adenoviruses used were deleted for E1 gene with CMV promoter driven firefly luciferase transgene inserted to this locus.

Directed mutagenesis by PCR was used to replace the KKTK motif in the 5/3 fiber shaft with the GAGA sequence, using pTU.5/3 as a template [54]. First, we created a 901 bp product with a forward primer (5E3F) 5′-gaaatcagctactttaatctaac-3′ (site in E3, before fiber) and a reverse primer (5GAGA) 5′-**ggctccggctcc**gagaggtgggctcacagtggttacattt-3′ (site in fiber shaft, repeat 3, including KKTK area, bold = replacing sequence for GAGA). Next, we used a forward primer (3GAGA) 5′-**ggagccggagcc**tcaaacataaacctggaaat-3′ (site in fiber shaft, repeat 4, including KKTK area, bold = replacing sequence for GAGA) and a reverse primer (3KR) 5′-aatcatcgctgaggagacca-3′ (site after knob region) resulting in a 2042 bp product. These fragments were combined with PCR SOEing using 5E3F and 3KR primers and thus the final 2932 bp PCR product contained the complete fiber gene, KKTK-mutation (GAGA) and Ad3 knob replacing the Ad5 knob. Homologous recombination with a *Swa*I-linearized rescue plasmid containing the complete adenovirus 5 genome was performed. Finally, a second homologous recombination between this plasmid and *Pme*I-linearized pShuttle. CMV-Luc resulted in a *E1*-deleted adenovirus 5 genome containing a GAGA mutated 5/3 chimeric fiber shaft and the luciferase transgene in a deleted *E1* region (pCMV.Luc.5/3S*). The Ad5/3lucS* genome was released by *Pac*I digestion and transfection to 293 cells was done for amplification and rescue.

All phases of the cloning were confirmed with PCR and multiple restriction digestions. The final 2932 bp PCR product was sequenced. Absence of wild type E1 and the presence of knob, fiber and GAGA mutation were confirmed with PCR. All adenoviruses were amplified on 293 cells and viruses were purified on two cesium chloride gradients, according to standard protocols. Absence of wild type contamination and presence of featured genes was confirmed by PCR with relevant primers. Concentration of viral particles (VP) was assessed by measuring absorption at 260 nm and the titer of plaque forming units (pfu) was assessed by TCID_50_ assays on 293 cells. All large scale virus production batches used were tested for the absence of endotoxin contamination.

### *In vitro* Transduction Assays

24-well plates were seeded with 50 000 (SKOV3.ip1 and Hey) or 100 000 (M4A4-LM3 and PC-3) cells/well. The following day cells were infected with 40, 200 or 1000 viral particles per cell in 200 µl of growth media with 2% FCS per well and incubated for 30 min at 37°C. Cells were then washed once and complete growth media was added. 24 h later cells were lyzed by 20 min incubation at room temperature with 200 µl per well of Luciferase Cell Culture Lysis Reagent (Promega, Madison, WI, USA) and lysates were then frozen to −80°C. Luciferase expression was analyzed at a later stage with the Luciferase Assay System (Promega). Experiments were carried out with triplicate wells.

HepG2 (100 000 cells per well) were seeded on 24-well plates and infected 24 h later with or without coagulation factors. Viruses were preincubated on a rocker at 4°C for 30 min with media containing 10 µg/ml human factor X or 5 µg/ml human factor IX (Haematologic Technologies Inc., Essex Junction, VT, USA), corresponding to physiological blood concentrations. 200 µl of mixture per well was added on HepG2 cells and incubated for 60 min at 37°C. Cells were then washed, incubated, lyzed and analyzed for luciferase expression as described above.

### Blocking Assays

To investigate the ability of recombinant Ad5 and Ad3 knobs to block transduction, infection with Ad5/3luc1 and Ad5/3lucS* was performed in the presence of the purified knob proteins [31]. 50 000 SKOV3.ip1 cells per well were seeded on 24-well plates. After one day, cells were washed and preincubated with increasing concentrations of Ad5 or Ad3 knob [31] in 100 µl of DMEM with 2% FCS for 15 min at room temperature. Virus was then added at 1000 VP/cell in 100 µl of 2% DMEM, followed by a 30 min incubation at room temperature. After washing, complete growth media was added and cells were incubated for 24 h at 37°C. Cells were then lyzed and luciferase activity determined as described above. In heparin blocking assays virus was preincubated for 45 min at 4°C with increasing concentrations of heparin (LEO Pharma, Malmö, Sweden) diluted in DMEM supplemented with 0.1 ng/ml bovine serum albumin. 200 µl of heparin-virus mixture per well was added on monolayers of SKOV3.ip1 cells for 30 min at room temperature. Cells were then washed, incubated, lyzed and analyzed as above.

### Animals and Warfarinization

Female Nude NMRI mice were obtained from Scanburn (Karlslunde, Danmark) at 3–4 weeks of age. All animals were acclimated for 2 weeks. For cell and virus injections and IVIS-imaging, mice were anesthetized with isoflurane (IsoFlo® vet, Orion Pharma, Turku, Finland). For tail vein blood sampling, mice were anesthetized with fentanyl citrate 0.5 mg/kg, fluanisone 15 mg/kg and midazolam 7.5 mg/kg mouse weight diluted in sterile water. To ablate coagulation factors, 133 µg warfarin (Waran®, Nycomed, Stockholm, Sweden) was administered subcutaneously in 100 µl of sterile NaCl 72 and 24 h before virus injection.

### Biodistribution Experiments

Mice were inoculated with 2×10^6^ M4A4-LM3 cells into the left and right uppermost mammary fat pads and tumors were allowed to develop until they reached a size of approximately 5 mm in diameter. Viruses were injected via the tail vein in 150 µl sterile NaCl. Control mice received NaCl only.

To determine the virus titer in tissues, mice were sacrificed 30 min or 3 h after injection. Whole blood was collected by cardiac puncture in heparinized Microtainer® collection tubes (#365952) and tumors and organs collected, snap frozen and stored at −80°C. Simultaneously, liver samples from the right lobe were fixed in neutral buffered 10% formalin for 24–48 h and routinely paraffin wax embedded for histological analysis. DNA was extracted from tissues and blood samples using QIAamp DNA mini kit (Qiagen, Helsinki, Finland). Quantitative real-time PCR was performed as described earlier, using primers and probe targeting the adenoviral E4 gene [8]. Mouse β-actin primers and probe served as internal control and to normalize viral DNA copies per amount of genomic DNA. For normalization on tumor samples, human β-actin primers and probe were used [55]. A regression standard curve for E4 copies was generated using adenoviral plasmid DNA serially diluted from 1×10^9^ copies to 1 copy. Standard curves for mouse and human β-actin were established, using known amounts (1800–0.18 ng and 800–0.08 ng) of DNA extracted from cultured cells. Cycle threshold values were plotted on the standard curves to determine the actual DNA copy number, and the number of adenoviral E4 copies per ng genomic DNA was subsequently calculated. Samples were run in duplicate wells.

For *in vivo* imaging, mice were injected intraperitoneally with 4.5 mg D-luciferin in 100 µl RPMI and imaged with the Xenogen IVIS imaging system (Perkin Elmer) and a charge-coupled device camera cooled to −120°C. Luminescence images with 1 sec exposure and photographic images were overlaid using Xenogen Living Image software (Perkin Elmer) and displayed in pseudo color, where the data values (photons) are made to correspond to various colors. Images were analyzed for luciferase expression using Igor image analysis software. For quantification of photon emission, regions of interest were drawn in the liver and tumor areas and photon emission from these regions was recorded.

For quantification of luciferase expression in tissues, mice were sacrificed 48 h after virus injection and tumors and organs collected and snap frozen to −80°C. Tissues were homogenized in 500 µl of Cell Culture Lysis Reagent (Promega) using a Tissue Master 125 (Omni, Kennesaw, GA, USA) tissue homogenizator and incubated 20 min at room temperature. Luciferase activity of lysates was quantified by the Luciferase Assay System (Promega) with TopCount (*PerkinElmer,* Waltham, MA, USA) plate reader luminometer. Relative light units (RLU) were normalized to total protein content of lysates, determined by Pierce® BCA Protein Assay Kit (ThermoSientific, Waltham, MA, USA).

### Histological Examination of Liver Tissues and Demonstration of Adenovirus Antigen

Consecutive 3–5 µm sections were prepared from the above described paraffin embedded liver samples and stained with hematoxylin-eosin (HE) and the Periodic Acid Schiff reaction (for assessment of hepatocellular glycogen content), and underwent immunohistology for the demonstration of adenovirus antigen.

For immunohistology, the avidin biotin complex peroxidase method was used. Briefly, after deparaffination through graded alcohols, endogenous peroxidase was blocked by incubation in methanol with 0.5% H_2_O_2_. Slides were then incubated in citrate buffer pH 6.0 (30 min at 96°C, followed by 20 min at room temperature) for antigen retrieval. After blocking of non-specific binding with undiluted horse serum for 10 min at room temperature, sections were incubated at 4°C for 15–18 h with the primary antibody (goat anti-gradient purified, disrupted Adenovirus type 5 virions) (ViroStat; 50 µg/ml in TBS with 0.05% Tween 20). Sections were then washed with TBS and incubated with biotinylated horse anti-goat IgG (Vector BA-9500) and ABC reagent (Vector PK-4000), followed by visualisation with diaminobenzidintetrahydrochloride (DAB) and Papanicolaou’s hematoxylin counterstain. Negative controls were incubated with a non-reactive antibody or TBS with 0.05% Tween 20 instead of the primary antibody.

The extent of virus antigen expression in hepatocytes was semi-quantitatively assessed, using the following scoring scheme: 0 - no positive hepatocytes; 1 - a few random disseminated positive hepatocytes; 2 - disseminated positive hepatocytes and some periportal patches of positive hepatocytes; 3 - disseminated positive hepatocytes and several small or some large periportal patches of positive hepatocytes; 4 - disseminated positive hepatocytes and several large periportal patches of positive hepatocytes.

### Amount of Virus Copies in Liver Parenchymal and Non-parenchymal Cells

Nude NMRI mice carrying above described mammary fat pad tumors were injected into the tail vein with 4×10^10^ VP of Ad5/3luc1, Ad5/3lucS* or Ad5luc1 with or without a pretreatment with warfarin. After 3 h, mice were sacrificed and livers were collected to separate the parenchymal cell (PC) and non-parenchymal cell (NPC) populations, using an approach similar to those previously described [25]. Briefly, excised livers were rinsed with NaCl to remove blood, sliced to smaller pieces and submerged briefly in HEPES buffer pH 7.2. Liver slices were then transferred to pH 7.5 HEPES buffer with 5 mM CaCl_2_ and 2 mg/ml collagenase and incubated for approximately 1 h at 37°C on a rocker. Cells were dispersed by gentle stirring in cold Hank’s-HEPES buffer containing 0.1% BSA and filtered through cotton mesh sieves. Differential centrifugation was performed to separate PC and NPC populations. Total DNA was then extracted and quantitative PCR for adenoviral E4 and mouse β-actin was performed as above.

### Plasma Liver Enzymes and Serum Cytokines after Intravenous Administration

Blood samples were collected at 6 h after intravenous administration of 4×10^10^ VP of viruses or NaCl only to tumor bearing mice by puncturing the tail vein. Samples were left to clot at room temperature and then centrifuged twice at 2000 rcf for 10 min to separate serum. Cytokine levels in serum samples were measured using BD Cytometric Bead Array (CBA) Soluble Protein Master Buffer Kit and BD CBA mouse interleukin (IL)-6, monocyte chemoattractant protein (MCP)-1 and tumor necrosis factor (TNF) Flex sets. BD FACSArray bioanalyser, BD FACSArray System Software and FCAP Array software were used to read and analyze data (BD Biosciences, San Diego, CA USA).

Blood samples for liver enzyme and blood cell count analysis were drawn by cardiac puncture when animals were sacrificed 48 h after virus injection. Half the blood was collected in heparinized Microtainer® collection tubes (Becton Dickinson, Helsinki, Finland) and plasma was separated by centrifuging samples at 2000 rcf for 10 min. The other half of the blood was collected in EDTA coated Microtainer® collection tubes (Becton Dickinson). ALT and AST levels in plasma and blood cell counts in EDTA anti-coagulated blood were quantified at the Laboratory of the Production Animal Hospital, Faculty of Veterinary Medicine, University of Helsinki by routine methods.

### Statistical Analysis

Statistical analyses for *in vitro* transduction assays were conducted with ANOVA and Bonferroni multiple comparisons test and Dunnet’s t-test. Effect of coagulation factors *in vitro*, *in vivo* imaging data and cytokine, liver enzyme and blood cell count data were analyzed with the two tailed Student’s t-test. Tissue luciferase expression and DNA contents were analyzed with ANOVA with the Bonferroni multiple comparisons test and the Dunnet’s t-test.

## Results

### *In vitro* Cancer Cell Transduction Capacity is not Severely Impaired by Mutation of the KKTK Motif

**Figure 1:**
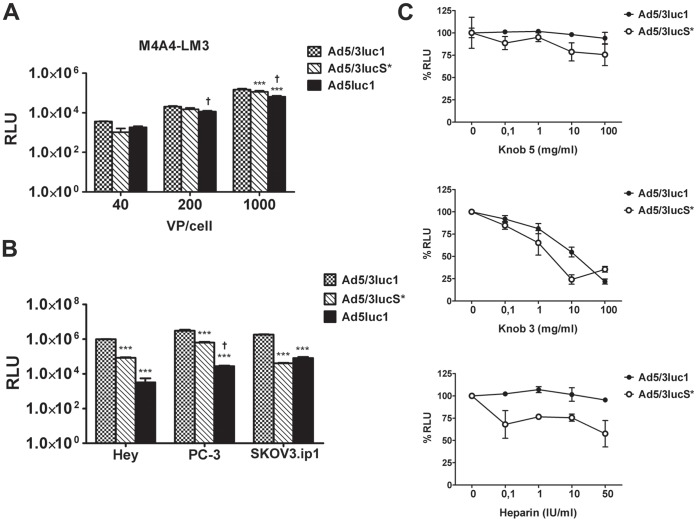
Cancer cell transduction in comparison to Ad5 is retained despite KKTK motif mutation of the Ad5/3 shaft. To study the cell transduction properties of the mutated virus A) M4A4-LM3 human breast ductal carcinoma cells were infected with indicated viruses at 40, 200, and 1000 viral particles (VP) per cell. B) Hey ovarian adenocarcinoma, PC-3 prostate cancer, SKOV3.ip1 ovarian adenocarcinoma cells were infected with 200 VP/cell. Unbound virus was removed after 1 h incubation and luciferase activity was measured from cell lysates after 24 hours of incubation. C) SKOV3.ip1 cells were preincubated with indicated concentrations of free recombinant knob 5 or knob 3 proteins and thereafter infected with 1000 VP/cell of Ad5/3luc1 or Ad5/3lucS* alone or virus preincubated with indicated concentrations of heparin. Luciferase transgene activity was quantified with a luminometer and expressed as relative light units (RLU) per ml of cell lysate. Assays were performed in triplicates, expressed as mean+SD. A–B) *** *p*<0.001 against Ad5/3luc1, ^†^ *p*<0.01 Ad5luc1 vs. Ad5/3lucS*.

In transduction assays on human breast cancer cells, Ad5/3lucS*transduced M4A4-LM3 cells efficiently as measured by luciferase expression from cells 24 h after infection (Fig 1A). At 40 VP/cell and 200 VP/cell, Ad5/3lucS* infection resulted in similar gene expression as Ad5/3luc1, whereas at 1000 VP/cell gene expression by Ad5/3luc1 was 1.3-fold higher(p<0.001). When compared with Ad5luc1, Ad5/3lucS* infection resulted in similar luciferase activity at 40 VP/cell but at a significantly higher level of transgene expression with higher titers (p<0.01) (Fig 1A). In other human cancer cell lines, Ad5/3lucS* gene expression was decreased 5- to 46-fold compared to Ad5/3luc1 (*p*<0.001), but was similar or up to 25-fold higher than Ad5luc1 (*p*<0.001 for PC-3) (Fig. 1B).

### Binding Properties of the knob are Retained in the KKTK Mutated Chimeric Adenovirus

Ad5/3luc1 transduction has been shown to be blocked by preincubation of cells with free recombinant knob3 but not knob 5, indicating that gene transfer involves interaction between the knob and cellular receptors [31]. To study whether the mechanism of knob interactions was similar for Ad5/3luc1 and Ad5/3lucS*, the knob blocking experiments were performed on SKOV3.ip1 cells. Free knob 5 was unable to block either Ad5/3luc1 or Ad5/3lucS*, whereas free knob 3 blocked gene expression by both Ad5/3luc1 and Ad5/3lucS*similarly in a dose dependent manner (Fig. 1C).

It has previously been shown that Ad3 binding to cells is not inhibited by heparin, an analog for heparin sulphates, while Ad5 binding is blocked by heparin in a dose dependent manner [18]. Although heparin did not affect Ad5/3luc1 cell transduction, it reduced Ad5/3lucS* gene expression up to 24% at 10 IU/ml (*p* = 0.044) and up to 42% at the highest heparin concentration (*p* = 0.001) (Fig. 1C).

### Coagulation Factors Cannot Compensate for Reduction of Transduction by Ad5/3lucS* in Hepatic Cells

**Figure 2:**
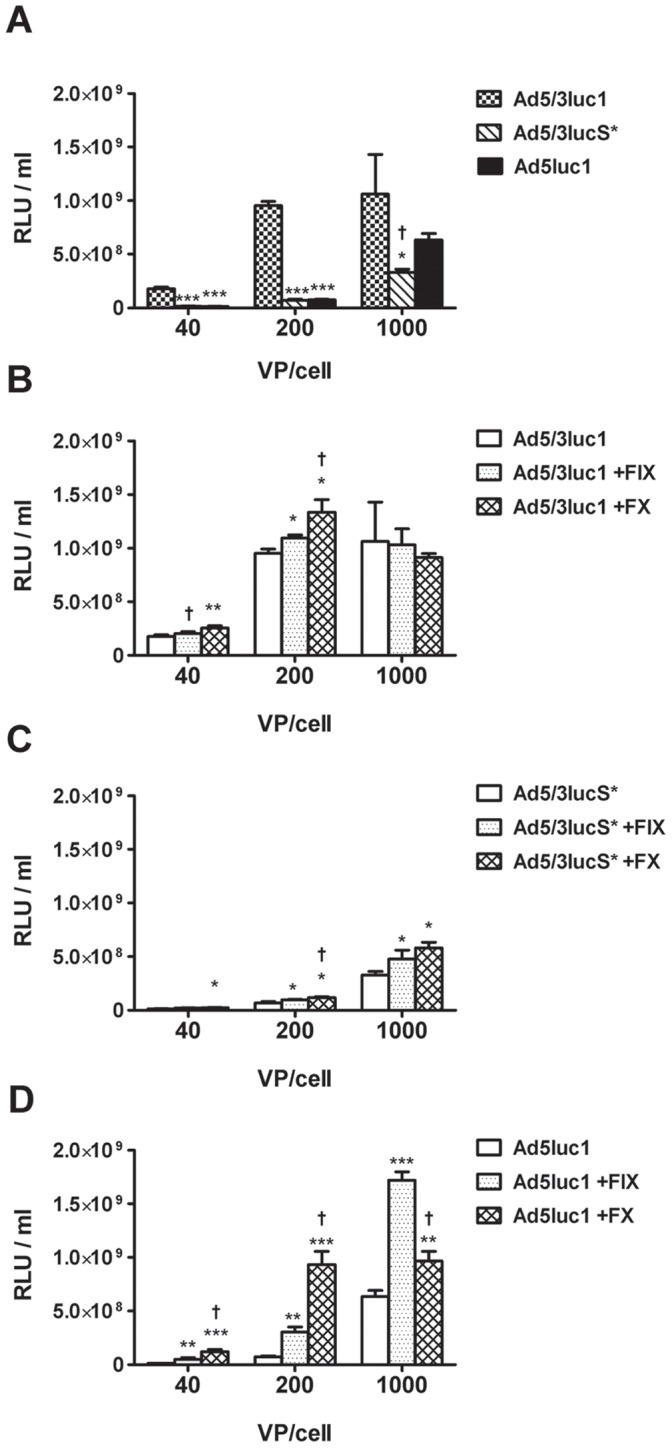
KKTK mutated 5/3 virus is detargeted from human hepatocytes but coagulation factors result in enhanced transduction. A) Monolayers of HepG2 cells were infected with increasing doses of Ad5/3luc1, Ad5/3lucS* or Ad5luc1 and luciferase expression was quantified 24 hours later. * *p*<0.05, *** *p*<0.001 vs. Ad5/3luc1, ^†^ *p*<0.05 Ad5luc1 vs. Ad5/3lucS*. B–D) HepG2 cells were infected with either virus only or virus preincubated with physiological concentrations of factor X (FX) or factor IX (FIX) and luciferase activity was measured 24h later. * *p*<0.05, ** *p*<0.01, *** *p*<0.001 vs. virus only, ^†^ *p*<0.05 vs. virus+FIX. Assays were performed in triplicates, expressed as mean+SD.

Coagulation factors IX and X (FIX and FX) have been implicated in cell transduction by adenoviruses, particularly of hepatocytes. FIX has initially been shown to bind the Ad5 fiber knob to “bridge” virus entry into cells [17] and later FX has been shown to bind to Ad5 hexon hypervariable regions with high affinity [47]. We therefore investigated the interaction of these factors with the KKTK mutated virus. HepG2 cells were infected in the presence or absence of FIX and FX and viral gene expression was measured in cell lysates 24 h later (Fig. 2). In the absence of coagulation factors, hepatocyte transduction by Ad5/3lucS* was lower than by the other viruses (*p*<0.05) (Fig. 2A). At 40 and 200 VP/cell infection, both FIX and FX enhanced cell transduction of Ad5/3luc1 by approximately 15% and 50%, respectively (Fig. 2B). When infecting cells with 1000 VP/cell, Ad5/3luc1 transduction seemed to saturate, as preincubation with coagulation factors did not enhance gene expression. For Ad5/3lucS*, FIX and FX improved cell transduction by 40% and 70% on average, but gene transfer was not restored to the levels of Ad5/3luc1 (Fig. 2C). Similarly, Ad5luc1 transduction was enhanced with both FX and FIX, by 700% and 260% on average, respectively (Fig. 2D). The effect of addition of coagulation factors was more pronounced for Ad5luc1 than for either Ad5/3luc1 or Ad5/3lucS* (Fig. 2).

### KKTK Mutation and Warfarinization both Reduce Viral Gene Expression in the Liver

**Figure 3:**
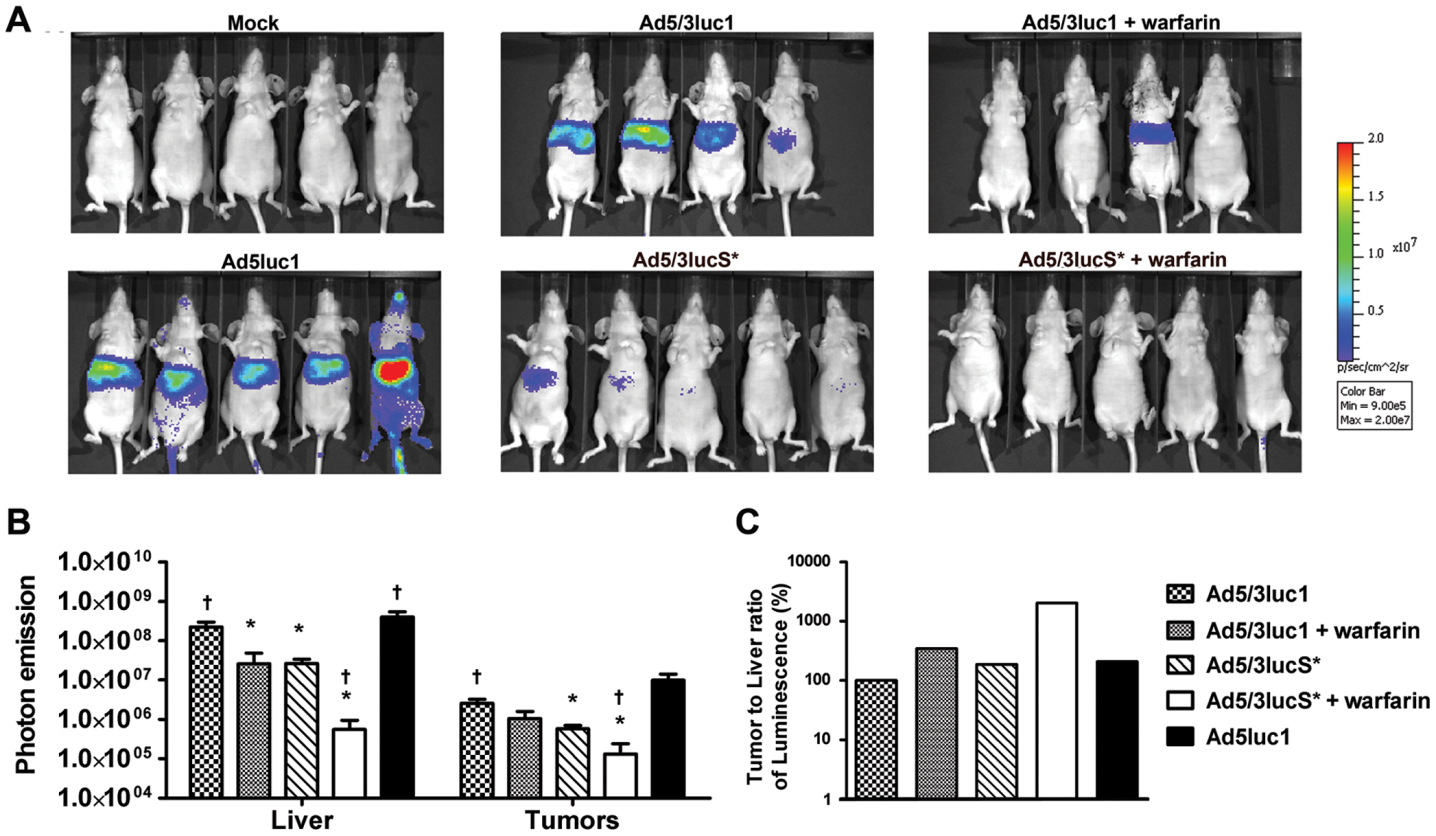
Warfarinization enhances liver detargeting of Ad5/3lucS*. Nude NMRI mice carrying M4A4 -LM3 mammary fat pad tumors were treated with 4×10^10^ VP of Ad5/3luc1, Ad5/3lucS* or Ad5luc1 or NaCl only to the tail vein. Indicated groups had been pretreated with warfarin to deplete vitamin K-dependent coagulation factors. A) 24 hours later mice were imaged for transgene activity by injecting D-luciferin i.p. and IVIS luminosity imaging. B) Regions of interest were drawn around liver and tumor areas to quantify photon emission signals and mock was subtracted. C) Ratio of luciferase expression quantified from tumors and livers was calculated. KKTK mutation reduces liver transduction to similar degree as warfarin. Both also improve the ratio of tumor to liver transduction, best result with Ad5/3lucS*+warfarin. Mean+SD, n = 4–5 mice per group. * *p*<0.05 vs. Ad5/3luc1, ^†^ *p*<0.05 vs. Ad5/3lucS*.

**Figure 4:**
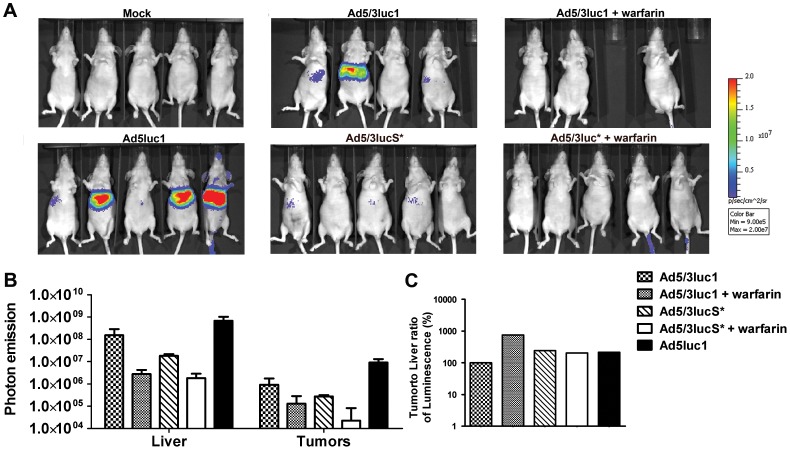
Warfarinization and KKTK mutation both result in reduced liver gene expression. Nude NMRI mice carrying M4A4 -LM3 mammary fat pad tumors were treated with 4×10^10^ VP of Ad5/3luc1, Ad5/3lucS* or Ad5luc1 or NaCl only to the tail vein. Indicated groups had been pretreated with warfarin to deplete vitamin K-dependent coagulation factors. A) 48 hours later mice were imaged for transgene activity by injecting D-luciferin i.p. and IVIS luminosity imaging. B) Regions of interest were drawn around liver and tumor areas to quantify photon emission signals and mock was subtracted. C) Ratio of luciferase expression quantified from tumors and livers was calculated. KKTK mutation and warfarinization reduce liver transduction and improve the ratio of tumor to liver transduction, best result with warfarinization. ^†^ *p*<0.05 vs. Ad5/3lucS*.

To study the effect of both the KKTK mutation and warfarinization on gene expression *in vivo,* mice were imaged with IVIS for luciferase activity 24 and 48 h after intravenous administration of virus (Fig. 3A, Fig 4A). At 24 h, mice that received Ad5/3lucS* had lower levels of luciferase expression in the liver compared to those that received Ad5/3luc1 and Ad5luc1 (*p*<0.05) (Fig. 3A,B). A similar reduction in liver transduction was observed in mice pretreated with warfarin to deplete vitamin K–dependent coagulation factors (*p*<0.05). The best result in terms of liver detargeting was obtained using Ad5/3lucS* in warfarinized mice, which resulted in a400-fold reduction of gene expression compared to Ad5/3luc1 (*p*<0.05) (Fig. 3B). In M4A4-LM3 tumors, gene expression of Ad5/3lucS*as measured by IVIS was approximately 10-fold lower compared to Ad5/3luc1 (*p*<0.05) and Ad5luc1 (*p* = 0.063) (Fig. 3B). The combination of warfarinization with Ad5/3luc1 resulted in similar degree of luciferase expression in tumors as Ad5/3luc1 alone, whereas warfarinization prior to Ad5/3lucS* administration led to lower luciferase expression that with either Ad5/3luc1 or Ad5/3luc* alone (*p*<0.05). This suggests that coagulation factors play a role in tumor transduction with Ad5/3lucS*, but not with Ad5/3luc1.

Liver is the major organ of accumulation of viral particles after systemic delivery [6], [7], [8], [9]. In contrast, anti-tumor efficacy is determined by transduction of the tumor [56]. However, the intravenous dose cannot be increased in an unlimited fashion to achieve sufficient tumor transduction, due to toxicities triggered by the vector [11], [12], [16]. Therefore, increasing the tumor to liver transduction ratio could result in less hepatic adverse events while retaining tumor transduction. Alternatively, an improved ratio might allow increasing the dose for improved tumor transduction while retaining the same level of liver toxicity. Interestingly in the IVIS imaging, the tumor to liver ratio of gene expression of Ad5/3luc1 and Ad5/3lucS* were 3.5- and 20-fold higher, respectively, after warfarinization than with virus alone, and 2-fold higher with Ad5/3lucS* alone compared to Ad5/3luc1 (Fig. 3C). The results of IVIS imaging at 48 h after injection showed similar trends, except here the tumor to liver ratio was highest in the Ad5/3luc1+warfarin group (Fig. 4).

**Figure 5:**
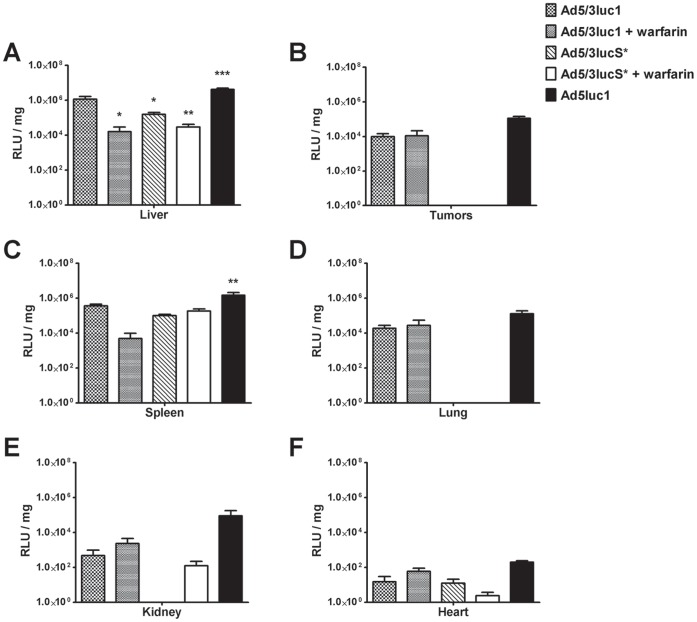
Hepatic luciferase expression is reduced with both KKTK mutation and warfarinization. A–F) Nude NMRI mice carrying mammary fat pad tumors were treated intravenously with 4×10^10^ VP of Ad5/3luc1, Ad5/3lucS* or Ad5luc1. Some groups were pretreated with warfarin to deplete coagulation factors. 48 hours after virus injection mice were sacrificed and luciferase expression in homogenized tissue lysates was quantified and normalized for total protein content of lysates. KKTK mutation reduced hepatic transgene expression, but reduction was also seen in tumors and other tissues. Mean+SD, n = 3–5 mice per group. *p<0.05, **p<0.01, ***p<0.001 vs. Ad5/3luc1.

Luciferase assays performed on tissue lysates excised from mice at 48 h yielded similar results as the IVIS imagings, with some exceptions (Fig. 5). Hepatic expression of luciferase was approximately 70-fold lower after warfarinization (*p*<0.05), 10-fold lower with the KKTK mutated virus (*p*<0.05) and 40-fold lower with their combination (*p*<0.01), when compared to Ad5/3luc1 (Fig. 5A). Ad5luc1 resulted in 4-fold higher hepatic and splenic luciferase levels than the capsid chimeric Ad5/3luc1 (*p*<0.01). In the spleen, warfarinization reduced transduction by approximately 70-fold, but this was not statistically significant (Fig. 5C). For other tissues, capsid mutations or coagulation factor depletion did not lead to significant differences although there was a strong trend of lower tumor, lung, kidney and also heart transduction with Ad5/3lucS* as compared to Ad5/3luc1 (Fig. 5B, 5D-5F). Interestingly, although only a minor reduction in tumor transduction and even increases in tumor-to-liver ratio were observed by IVIS imaging, in the tissue homogenate assay transgene activity in Ad5/3lucS* treated tumors was unmeasurable (*p* = 0.51).

### No Major Differences in the Distribution of Virus Particles after Injection

**Figure 6:**
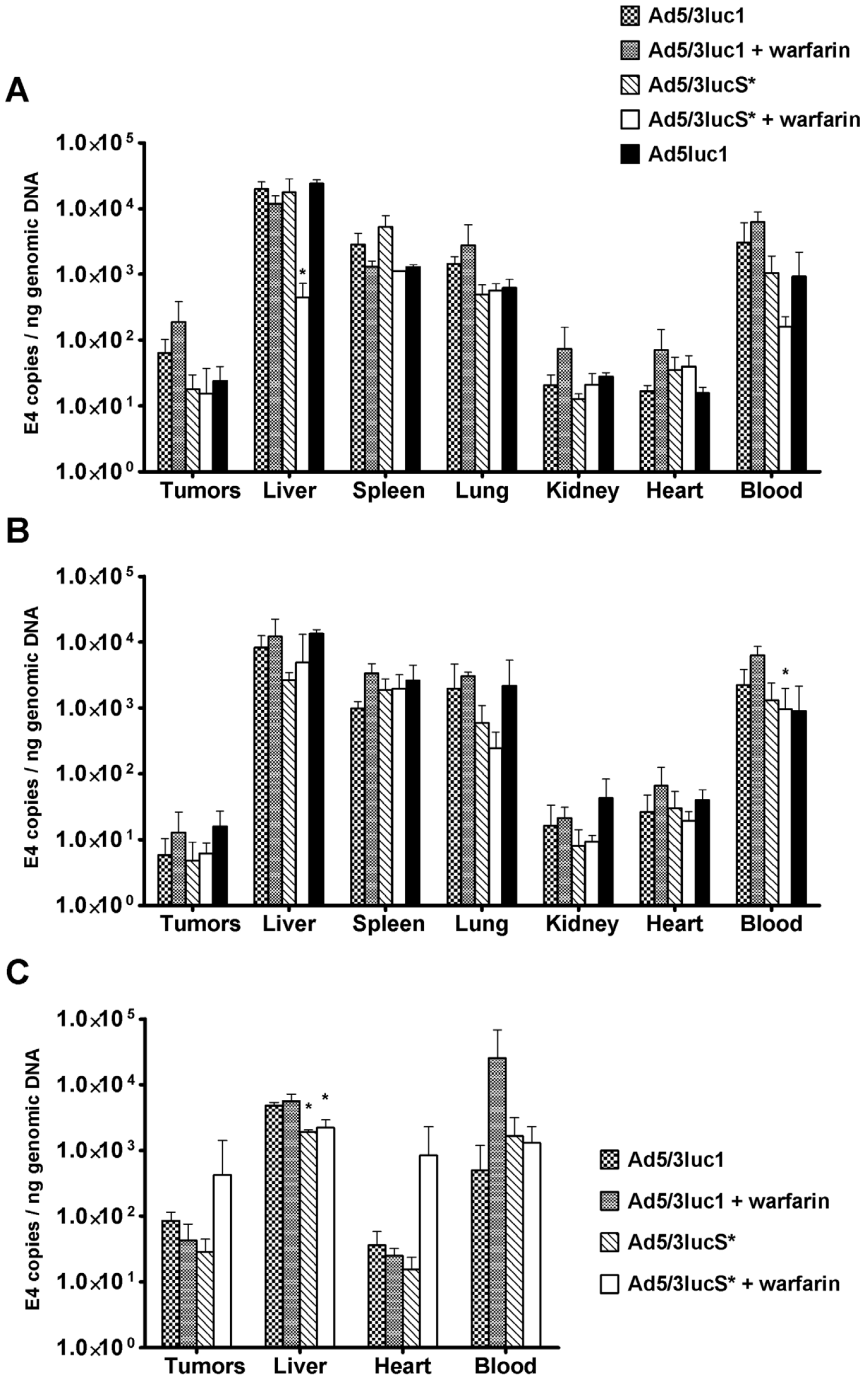
Distribution of viral particles is not affected by KKTK mutation at lower viral dose or warfarinization. Mice carrying xenograft mammary fat pad tumors were injected with 4×10^10^ VP of Ad5/3luc1, Ad5/3lucS* or Ad5luc1 into the tail vein. Indicated groups had been pretreated with warfarin. Mice were sacrificed A) 30 minutes or B) 3 hours after virus injection and organs, tumors and whole blood was collected. Viral loads in the samples were quantified by adenoviral E4 region qPCR. Mouse β-actin for organs and blood and human β-actin for tumor tissue was used to normalize viral titers to genomic DNA. At 30 minutes there was less liver uptake with Ad5/3luS*+warfarin, this difference disappeared by 3 hours. C) At a higher dose of 5×10^10^ VP (analysis at 30min), there was a significant decrease in liver uptake of Ad5/3lucS* virus with or without warfarin compared to Ad5/3luc1, and a trend for higher tumor uptake in the Ad5/3lucS* virus with warfarin group. Mean+SD, n = 3 mice per group. *p<0.05vs.Ad5/3luc1.

Previous reports have described that there is not always a correlation between biodistribution of virus particles and transgene expression with capsid modified viruses [8], [23], [25]. One reason is that some cell types, such as macrophages in the liver and spleen, do not allow transgene expression even though they have a major role in virus uptake. Also, reduced entry may result in increased blood persistence, and therefore the blood content of organs could play a role in genome to transgene expression ratios. Thus, it is of relevance to study not only the transgene expression but also the presence of viruses. To investigate this, we collected organs, tumors and blood 30 min and 3 h after virus injection and measured viral copy numbers by real-time PCR. At 30 min, viral particle amounts were similar between the groups in nearly all tested tissues. The only noted differences were that there was less virus in the livers of the Ad5/3lucS*+warfarin group and a trend of less virus in the Ad5/3lucS*+warfarin blood samples and more virus in tumors of Ad5/3luc1+warfarin treated mice (Fig. 6A). At 3 h, there were no major differences, except minor variation in blood titers (Fig. 6B). The transient reduction in liver and blood titers in the Ad5/3lucS*+warfarin group may reflect differences in circulatory kinetics and blood, as blood collected by cardiac puncture mainly collects blood from large vessels.

To assess the effect of dose on viral particle distribution we conducted an additional experiment with Ad5/3luc1 and Ad5/3lucS* injected at a slightly higher dose (5×10^10^ VP intravenously) and collected tissues 30 min after injection. Interestingly, we detected approximately 2-fold more virus in livers with Ad5/3luc1 infection compared to Ad5/3lucS* with or without pre-warfarinization. In contrast, virus particle amounts in tumors were similar (Fig. 6C).

### Virus Administration does Lead to Virus Uptake by Kupffer Cells and Hepatocytes, but does not Lead to Significant Pathological Changes at Early Time Points

**Figure 7:**
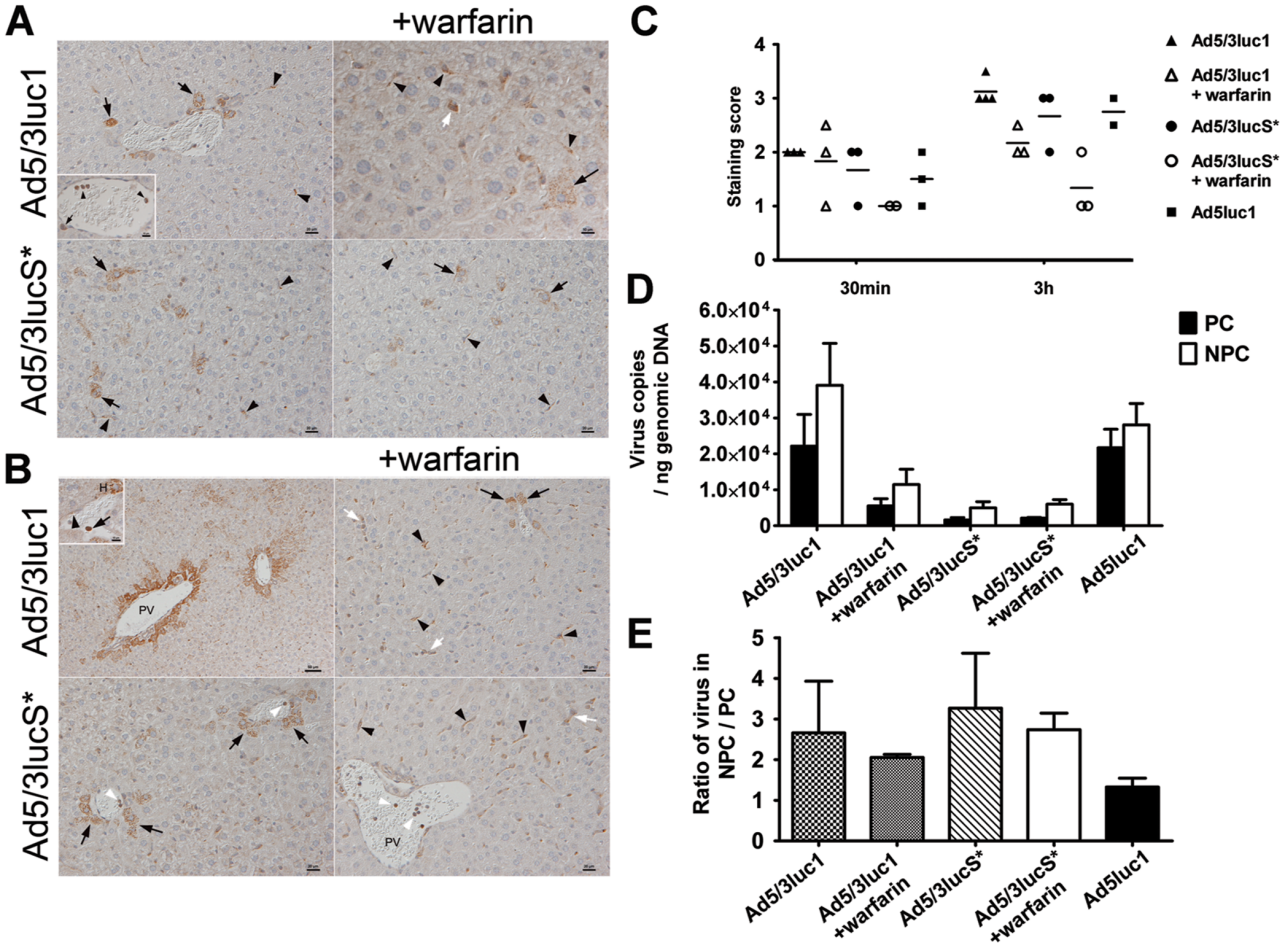
Subtle differences are observed in viral antigen expression pattern and DNA copy numbers in the liver. Mammary fat pad tumor carrying mice were injected intravenously with 4×10^10^ VP of the indicated viruses or NaCl only. The indicated groups had been pretreated with warfarin to deplete coagulation factors. Immunohistology for adenovirus hexon antigen expression in the liver A) 30 min after virus administration. Viral antigen (stains brown) is observed within Kupffer cells (arrowheads) and in individual hepatocytes (black arrows) and occasional leukocytes in sinus (white arrow). Inset: Viral antigen can also be seen in neutrophils in the circulating blood in a central vein (arrowheads) and in an adjacent sinus (arrow). Note the generally weak antigen expression and its restriction to Kupffer cells (arrowheads) and some random hepatocytes (arrows) in an Ad5/3lucS*+warfarin treated mouse. Bars = 20 µm for Ad5/3luc1 and Ad5/3lucS* and Ad5/3lucS*+warfarin, Bars = 10 µm for inset and Ad5/3luc1+ warfarin. B)3h after virus administration. Viral antigen expression in Kupffer cells (arrowheads), hepatocytes (arrows), some leukocytes in sinuses (white arrows) and occasional neutrophils within portal veins (white arrowheads). PV = portal vein. Inset: Portal vein. Viral antigen expression in hepatocyte (H), neutrophil (arrow) and endothelial cell (arrowhead). Note the strong antigen expression in an Ad5/3luc1 treated mouse in hepatocytes surrounding portal areas and the weak antigen expression with restriction to Kupffer cells in an Ad5/3lucS*+warfarin treated mouse. Bar = 50 µm for Ad5/3luc1, Bars = 20 µm for Ad5/3luc1+warfarin, Ad5/3lucS* and Ad5/3lucS*+warfarin, Bar = 10 µm for inset. C) Semi-quantitative assessment of virus antigen expression in hepatocytes, n = 2–4 mice/group. D-E) Mice were sacrificed 3 h after virus administration and cells of the liver were freshly isolated and separated into liver parenchymal cells (PC; hepatocytes) and non-parenchymal cells (NPC; include Kupffer cells and endothelial cells). D) DNA was extracted and the amount of virus particles determined by quantitative PCR with primers and probes targeting the adenoviral E4 region. Primers and probes for mouse β-actin were used to normalize viral titers to sample genomic DNA. E) The ratio of virus present in non-parenchymal versus parenchymal cells was calculated. D-E) Mean+SD, n = 3–4 mice per group.

In order to assess the potential immediate cytopathic effect and the uptake and distribution of virus in the early phase after administration, we undertook a histological examination and performed immunohistology for adenovirus antigen on liver sections of mice 30 min and 3 h after Ad5luc1, Ad5/3luc1 and Ad5/3lucS* administration. We did not observe any distinct pathological changes at both time points. However, we observed variable random or periportal hepatocellular glycogen loss in a few mice in Ad5luc1 treated mice at both time points and in both Ad5/3luc1 and Ad5/3lucS* treated mice at 3 h post injection with and without warfarinization (data not shown). These changes indicate energy loss and early degeneration. Virus antigen was consistently detected in Kupffer cells, but was also present in variable numbers of hepatocytes in all groups at both time points. At 30 min post injection, positive hepatocytes were generally sparse and found randomly distributed (Fig. 7A). At 3 h, positive hepatocytes were mainly seen around portal areas where they were arranged in a circular manner (Fig. 7B). At both time points, the number of positive hepatocytes appeared particularly low in livers from animals that had been treated with Ad5/3lucS*+warfarin (Fig. 7A, B). In addition, neutrophils in blood vessels and occasional endothelial cells were found to express viral antigen (Fig. 7A, B). The extent of hepatocellular adenovirus antigen expression is graphically illustrated in Fig. 7C.

We then investigated whether mutation of the KKTK region or pre-warfarinization would affect virus uptake into liver parenchymal cells (eg. hepatocytes) and/or non-parenchymal cells (including Kupffer and sinusoidal endothelial cells). For Ad5, transduction of hepatocytes is thought to be a receptor-mediated process, whereas uptake by Kupffer cells is receptor independent [57]. Warfarinization, mutation of the KKTK motif and their combination all resulted in a trend of lower viral amounts in parenchymal and non-parenchymal cells (Fig. 7D). The ratio of amount of virus in non-parenchymal versus parenchymal cells was slightly higher with Ad5/3lucS* compared to Ad5/3luc1 and slightly, but not significantly, lower with warfarinization for both of these viruses (Fig. 7E).

### Ad5/3lucS* Results in Attenuated Cytokine Response

**Figure 8:**
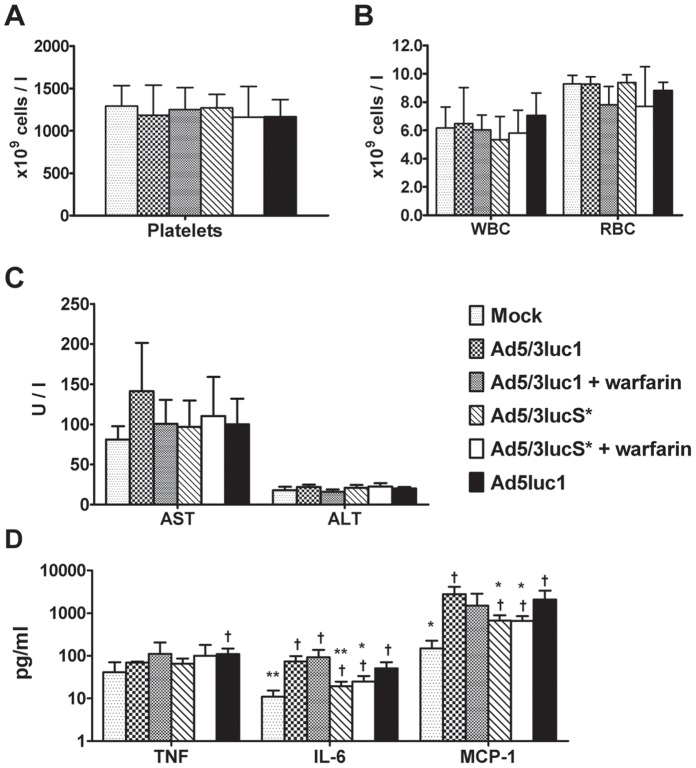
Blood cell counts and liver enzymes after intravenous virus administration. Mammary fat pad tumor carrying mice were injected intravenously with 4×10^10^ VP of the indicated viruses or NaCl only for mock, through the tail vein. Indicated groups had been pretreated with warfarin to deplete coagulation factors. Blood samples were collected 48 hours after injection. A) White blood cell (WBC) and red blood cell (RBC) and B) platelet counts were measured. C) Aspartate amino transferase (AST) and alanine amino transferase (AST) levels determined from plasma samples. D) Serum samples were collected 6 hours after treatment and interleukin (IL)-6, MCP-1 and TNF-alpha levels determined by FACSArray. Mean+SD, n = 2–5. Neither warfarinization nor KKTK mutation affected circulatory amounts of blood cells and the given virus treatments did not provoke liver enzyme elevations, but KKTK mutation resulted in milder cytokine responses. Mean+SD, n = 2–5 mice per group, * *p*<0.05, **p<0.01 vs. Ad5/3luc1, ^†^ *p*<0.05 vs. mock.

To investigate the effects of these modifications on the toxicity profile of intravenous virus, we analyzed cytokine responses, blood cell counts and liver transaminase levels. White blood cell, red blood cell and platelet counts were determined 48 h after virus administration and there were no changes in these parameters between the groups (Fig. 8). In addition, neither ALT nor AST levels were significantly elevated in any treatment group (Fig. 8). Serum TNF levels were significantly elevated only in the Ad5luc1 group (*p*<0.05 when compared to mock treated mice) and there were no significant differences in other treatment groups (Fig. 8). Serum IL-6 and MCP-1 were elevated in all treatment groups, but both were approximately 4-fold lower in mice treated with Ad5/3lucS* compared to Ad5/3luc1 (p<0.05), which could be indicative of higher safety. Warfarinization did not affect cytokine responses (Fig. 8).

## Discussion

Mutations of the KKTK region of the Ad5 fiber have been reported to result in effective liver detargeting. They have not been previously studied in the context of Ad5/3 chimeric viruses, which have shown considerable advantages over serotype 5 viruses in many aspects. In this study our aim was to produce an adenoviral vector genetically detargeted from the liver, but with retained transduction capacity of tumor tissues, by using the 5/3 knob chimeric adenovirus backbone with the KKTK mutation. Further, we sought to investigate the liver detargeting properties of the constructed virus in comparison to those of coagulation factor depletion and the combination of these. We found that ablation of the KKTK region resulted in a similar degree of reduction in liver transduction as the ablation of vitamin K dependent coagulation factors. Cancer cell transduction was retained *in vitro* with the chimeric KKTK mutant virus, but *in vivo* transgene expression in tumors was clearly decreased. This latter was not seen with coagulation factor ablation.

The 5/3 knob chimeric Ad5/3lucS* carrying a KKTK mutation infected various cancer cell lines in culture with reasonable efficiency. *In vitro* gene transfer by Ad5/3lucS* was not hampered compared to Ad5luc1, but it was slightly reduced in most cancer cell lines compared to the parental chimera Ad5/3luc1. This demonstrates that in combination with the5/3 chimeric fiber the KKTK mutation does not seriously impair the functionality of the fiber and the infection capability of the virus *in vitro*. Therefore, receptor binding *via* Ad3 knob seems less dependent on fiber flexibility than *via* the native Ad5 knob. In transduction blocking assays, free knob5 proteins were not able to block transduction of either Ad5/3luc1 or Ad5/3lucS*, whereas free knob 3 proteins blocked both similarly in a dose dependent fashion. Thus, the basic characteristics of the knob mediated binding - seen for Ad5/3luc1 - were retained in Ad5/3lucS*, demonstrating that the KKTK mutation does not interfere with knob-receptor interactions.

It has been proposed that heparin blocks transduction with Ad5 but not Ad3 [18]. In our assay, Ad5/3luc1 was not blocked by heparin, which is compatible with data reported for Ad3. Surprisingly, transduction of Ad5/3lucS* was reduced by high heparin concentrations, although the KKTK mutation is thought to abolish the HSPG binding by the shaft. Our observation could result from HSPG degradation by heparin. It has been proposed that in the wild type – short shafted - Ad3 interaction of the Ad3 knob with its receptor(s) requires also HSPGs as co-receptors [41]. In our assay heparin did not block Ad5/3luc1 transduction, which implies that HSPG may not be as necessary for the knob-receptor interactions of the Ad5/3luc1 chimera where the shaft is bent, as compared to fully serotype 3 Ads. Conversely, the straight-shafted Ad5/3lucS* appears to be more similar to the Ad3, as degradation of HSPG by heparin reduces gene delivery and therefore Ad3 knob mediated cell transduction through HSPG co-binding is reduced. Alternatively, as it has never been directly shown that KKTK is definitely the binding site for HSPG or heparin, it is possible that the mutation does not actually abolish an HSPG binding site but exerts its effects through alternate mechanisms related for example to three-dimensional conformational structure of the capsid. Yet another possible explanation for this observation could be a change in the pH of the media due to high heparin concentrations: The mutated virus might be more sensitive to pH changes due to the altered capsid structure. However, heparin blocking assays have resulted in controversial findings also in previous studies. Originally, Dechecchi et al. described blockage of Ad5 and Ad2 infection by heparin [18]. Later, Di Paolo et al. studied heparin blocking with and without buffering with HEPES and were unable to block infection by neither native Ad5 nor viruses with long chimeric shafts lacking the KKTK motif [58]. The multiple actions and effects of heparin could underlie these ambiguities.

Gene transfer to human hepatic HepG2 cells was significantly lower with Ad5/3lucS* than with Ad5/3luc1. When FIX and FX was added, transduction with both viruses was enhanced. This suggests that the coagulation factor pathway and the KKTK-related pathway are separate non-compensatory cell entry mechanisms, a feature that would be expected due to the proposed binding sites of the factors [17], [47]. Interestingly, addition of FIX or FX prior to Ad5luc1 infection resulted in a greater degree of enhancement of transduction, which implies that the native Ad5 capsid more responsive to addition of coagulation factors than the chimeric Ad5/3 capsid. This finding is supported by a recent report, where an Ad5 based chimeric vector with Ad35 fiber was found insensitive to infectivity enhancement by FX, leading to the investigators’ conclusion that the fiber is a predominant determinant for cell entry [59]. Several other vectors with pseudotyped fibers have been shown to be sensitive to FX mediated infectivity enhancement, to various extents [60]. Therefore, the extent of influence of the fiber on FX mediated infectivity likely depends on the particular fiber used. Indeed, FX enhanced transduction with our Ad5/3 chimeric virus, although to a lesser extent than for Ad5, demonstrating that Ad3 fiber knob chimerism allows for FX mediated enhancement.

Coagulation factor ablation *via* warfarin treatment and KKTK mutation, separately and in combination, both reduced *in vivo* liver gene transfer. The *in vivo* imaging data for transgene expression implied potentiated detargeting by the combination. However, tissue gene expression quantification did not confirm this, which may be due to differences in sensitivity and specificity of the respective methods of quantification. Interestingly, regardless of significant reductions in liver transgene expression by both, the KKTK mutation and warfarinization, viral particle accumulation in tissues was not affected at earlier time points, namely 30 minutes and 3 hours after i.v. administration, and was only somewhat lower in an experiment with higher dose of viruses. This finding is in accordance with previous data by Nicol et al. who described reduced VP counts of Ad5 lacking the KKTK motif in liver and spleen 5 days, but not 1 hour after intravenous injection in rats [22]. With regard to warfarinization, our findings agree with a previous report where virus copy numbers in the liver remained unchanged at early time points in [49] and previously published data demonstrating reduced gene expression in the liver after ablation of coagulation factors [46], [48], [50].

Nicol et al. showed that lack of gene expression by KKTK mutated virus was not caused by poor cell binding but rather by retardation of post-internalization steps [22]. An alternative explanation for the discrepancy between viral particle accumulation and gene expression in the liver with KKTK motif lacking vectors might be that the KKTK mutation redirects virus entry from parenchymal to non-parenchymal cells, as suggested by Koizumi et al. [25]. To investigate this, we performed immunohistochemical staining for adenoviral hexon antigen and measured viral loads in isolated liver parenchymal and non-parenchymal cells. Indeed, both tests showed a trend of lower Ad5/3lucS* content in hepatocytes at both investigated early time points. Since total viral copy numbers in the liver were not reduced, this indicates that the virus may indeed be preferentially taken up by Kupffer cells which were the cells that most consistently exhibited viral antigen *in situ*. Accordingly, the qPCR results also showed a trend of relatively more virus DNA in non-parenchymal cells, although the difference was not significant. Therefore our observations support those by Koizumi et al. [25], and the difference between Ad5/3lucS* viral particle accumulation and gene transfer in liver seems to be caused by more virus accumulation in the non-parenchymal cells including Kupffer cells, which results in virus degradation rather than gene expression. With warfarinization, there was a trend for less virus antigen in hepatocytes after warfarinization, particularly at the 3 h time point. However, the ratio of viral DNA accumulation in parenchymal *versus* non-parenchymal cells was not affected radically. Therefore, with warfarinization the distribution of virus among different non-parenchymal cells, such as endothelial cells and Kupffer cells, may be relevant. Alternatively, ablation of coagulation factor binding through warfarin treatment may induce alterations in the post-internalization steps and these phenomena are not mutually exclusive.

The results of the histological examination at 30 min and 3 h post virus administration and the liver enzyme data collected at 48 h did not provide any evidence of major viral hepatotoxicity in any of the groups. Blood cell counts were also unaffected. The variable degree of hepatocellular glycogen loss observed in particular in periportal areas is indicative of hepatocellular energy loss and might reflect functional impairment or early degenerative changes which would only lead to apparent pathologic effects at a later time point post injection. Intriguingly, liver detargeting by KKTK mutation reduced inflammatory cytokine responses, whereas warfarinization did not. Thus it is possible that the KKTK mutation provides some safety advantages, but this should be confirmed in other experimental models.

Neither coagulation factor ablation nor the mutation of KKTK decreased delivery of viral particles to tumors or other organs. In IVIS imaging transgene expression by Ad5/3lucS* was significantly lower in tumor compared to Ad5/3luc1. However, as transgene expression by Ad5/3lucS* in the liver was decreased even more, the ratio of transgene expression in tumors relative to the liver was better with Ad5/3lucS* compared to Ad5/3luc1 in this assay. However, data from tumor, lung and kidney tissue homogenates indicated a clear trend of lower level of gene expression by the KKTK-mutated virus than by the control viruses. Indeed, in tumor homogenates transgene activity was non-measurable with Ad5/3lucS*. Sensitivity of luciferase assay in vivo versus in vitro could explain some of these differences. Nevertheless, there was major variation between the relationship of delivery of physical particles and transgene expression *in vivo* and *in vitro.* Even though the KKTK mutation on a 5/3 chimeric backbone was tolerated well with respect to gene expression in cancer cells *in vitro*, the system is more delicate *in vivo*. This is not surprising, since the expression of many molecules relevant for adenovirus infection, such as coagulation factors and HSPG, is difficult to reproduce *in vitro.* The observed reduction in gene expression regardless of efficient viral particle delivery also sheds light on the *in vivo* behavior of the fiber shaft mutated virus, perhaps suggesting the importance of post-entry steps in tumor cells, more rapid clearing from cells of the tumor microenvironment or some poorly understood *in vivo* factors. Nonetheless, as transgene activity was low in tumor homogenates, in our opinion the approach would benefit from further improvements. In contrast, tumor transgene expression was completely retained with coagulation factor ablation.

The results of the present study confirm previous findings of our group and Shashkova et al., with regard to increased tumor transduction and better antitumor efficacy in warfarinized mice [49]. However, a contrasting result was reported by Gimenez-Alejandre et al. who described nearly completely abolished tumor transduction following intravenous virus administration to warfarinized mice [61]. Therefore, while our results are encouraging, tumor transduction in combination with coagulation factor ablation needs be more widely investigated in various cancer models to determine the possible effect of this treatment.

As human DSG-2 is not expressed in mouse tissues, fully serotype Ad3 viruses cannot infect murine cells and therefore can only be studied in vivo in transgenic murine models. Our results here imply that the chimeric serotype 5 virus with knob from serotype 3 is indeed able to infect various murine tissues efficiently as demonstrated by equal viral particle accumulation in murine tissues compared to wild type Ad5 capsid, and also efficient transgene expression in these tissues. This is in line with previously published data on Ad5/3 chimeric viruses [8], [34], and suggests that DSG-2 is not needed for *in vivo* infectivity by Ad5/3 chimeric viruses. However, human adenoviruses are in many aspects species-specific and also distribution of receptors used by the Ad5/3 chimeras is likely different in mice and humans. Therefore, data from xenograft models must always be interpreted with caution and experimental models with more resemblance to the human would be preferable. Eventually, many aspects of distribution of viral vectors in humans may eventually require clinical data in order to be fully comprehended.

Taken together, our data on the Ad5/3lucS* virus indicate that chimeric Ad5/3 may be a more suitable backbone than native Ad5 for introducing a mutation of the KTKK region, as indicated by promising transduction capacity *in vitro*. However, even though viral particles were efficiently delivered to tumor tissue also *in vivo,* transgene expression was nearly absent. Therefore the KKTK mutation affected distribution of viral particles differently from gene expression, highlighting the impact of post-internalization steps of cell transduction and distribution of virus within different cell types of tissues.

Overall, our study suggests that escaping coagulation factor binding is an appealing method of improving gene transfer efficacy of systemic delivery of adenoviral vectors, and KKTK mutation as an alternative method does not provide advantages over this. Ablation of coagulation factors leads to a degree of reduction of liver transduction that is comparable with mutation of the KKTK region, whereas tumor transduction and transgene expression are retained more reliably with warfarinization. Even with the 5/3 chimeric backbone the KKTK mutated vector did not deliver efficient transgene expression to the target tumors. Warfarin is an easily translatable approach as it is used in millions of patients to prevent blood clotting. However, the same effect may be achievable through vector engineering. Vectors mutated in putative coagulation factor binding sites have been described and they do indeed detarget the liver efficiently [62], [63], [64], but whether distant tumors are transduced equally effectively with this type of vectors remains to be confirmed.
